# Resilience as a Mediator Between Interpersonal Risk Factors and Hopelessness in Depression

**DOI:** 10.3389/fpsyt.2020.00010

**Published:** 2020-02-28

**Authors:** Alberto Collazzoni, Paolo Stratta, Francesca Pacitti, Alessandro Rossi, Valeria Santarelli, Massimiliano Bustini, Dalila Talevi, Valentina Socci, Rodolfo Rossi

**Affiliations:** ^1^ Department of Biotechnological and Applied Clinical Sciences (DISCAB), University of L’Aquila, L’Aquila, Italy; ^2^ Department of Mental Health, ASL 1, L’Aquila, Italy; ^3^ Department of Mental Health, Psychiatric Service of Diagnosis and Treatment, ASL, Rieti, Italy; ^4^ Department of Systems Medicine, Tor Vergata University of Rome, Rome, Italy

**Keywords:** resilience, mediator, mediation, hopelessness, humiliation, adverse early family experiences, interpersonal risk factor, depression

## Abstract

Several studies investigated the role of resilience as a mediating factor for psychopathological phenotypes. The aim of the current study is to explore the putative role of resilience as a mediator between different vulnerability factors and depressive symptoms. One hundred and fifty patients with a major depressive disorder diagnosis have been evaluated on the basis of humiliation (Humiliation Inventory), adverse past family experiences (Risky Family Questionnaire), hopelessness (Beck Hopelessness Scale), and resilience (Resilience Scale for Adult) scores. A multiple regression analysis and a bootstrapping method were carried out to assess the hypothesis that resilience could mediate the relationships between these risk factors as predictors and hopelessness as a dependent variable. Our results show that resilience has a mediating role in the relationship between several risk factors that are specifically involved in interpersonal functioning and hopelessness. The main limitations of the study are the cross-sectional nature of the study, the use of self-report instruments, the lack of personality assessment, and the consideration of the resilience as a unique construct. The understanding of the mechanisms through which resilience mediates the effects of different interpersonal risk factors is crucial in the study of depression. In fact, future prevention-oriented studies can also be carried out considering the mediating role of resilience between interpersonal risk factors and depressive symptoms.

## Introduction

Depression is one of the most disabling mental disorders, showing a heterogeneous clinical presentation (1) and it is related to different risk factors such as biological, psychological, and environmental (2). The relationship between risk factors for depression is complex, involving several interconnected pathways which can eventually shape the clinical presentation and outcome of depressed patients (3, 4).

Risk factors for depression include negative interpersonal experiences, such as enduring humiliation and early exposure to a dysfunctional familial environment. Farmer and McGuffin (5) studied the relationship between humiliation and depression showing that humiliating events may have a role in the onset of depression. Indeed, humiliating experiences, such as discrimination, social isolation, and rejection could trigger a depressive episode (6, 7). In fact, the enduring fear of being humiliated is considered one of the pathogenic negative beliefs in depression (8). Moreover, early humiliating experiences, such as bullying and peer victimization, are associated with hopelessness, depression, and suicidality (9).

Early adverse family experiences are a major risk factor for depression (10). Growing up in families characterized by conflicts, violence, and a cold, unsupportive and neglectful parental style results in a higher probability of childhood trauma (11), disruption of psychosocial functioning (12), and depressive symptoms. Specifically, lack of a supportive family during childhood could prevent the development of positive beliefs about one’s own self, others, and the future, increasing the likelihood of hopelessness (13).

Hopelessness can be defined as the negative expectations about future situations and events which could involve the self and the others (14). People who have these negative expectations think they cannot solve their problems, they never reach their goals (14), and that there will be more negative than positive times in their future (15). Beck considers hopelessness as the third component of his cognitive negative triad of depression which already involves the self and the world/environment (16, 17). Hopelessness is often reported in depressed and schizophrenic patient’s cases (18).

Hopelessness can be related to the interpersonal dynamics, as an inadequate parenting style. In particular, the parents use parental tactics, such as guilty or withdrawal of affection to manipulate the child’s emotional and psychological state. As a consequence, children who live with these parents can believe that they have no personal control regarding school activities, and they consider negative events as uncontrollable and overwhelming (19, 20). Moreover, hopelessness can also be related to personality characteristic as emotional instability (21). In the previous case, we can define hopelessness as “learned helplessness” (19–21).

The above-mentioned risk factors could have a direct effect on depressive symptoms; however, they could indirectly affect depression acting through a third mediating variable such as resilience which refers to positive adaptation, or ability to maintain or regain mental health, despite experiencing adversity. Its nature is dynamic throughout the lifespan and interacts with major domains of life functioning, such as intimate relationships and attachment (22). Resilience represents a protective factor against the development of psychiatric symptoms acting as a mediator in the relationship between risk factors and depressive symptoms. Several cross-sectional studies analyzed the mediating role of resilience in the relationship between risk factors and depression, in both clinical (23) and non-clinical populations (24, 25). For example, stigma and avolition were found to be connected to depression by the mediation of resilience in a population of schizophrenic patients (23). The relationship between stress and anxiety and depression in a non-clinical sample is partially mediated by resilience (24). Moreover, the self-perceived competence, considered a personal resilience factor, has been found to be a mediator (but not a moderator) of the relationship between stressful life events and depression in a sample of 9-th grade students during a two-wave longitudinal study. In fact, the direct effect of negative events on depressive symptoms diminished after controlling for self-perceived competence (25). However, resilience has been also considered as a moderator of the effect of risk factors on depressive symptoms. For example, resilience has shown a moderating effect on depressive symptoms severity in individuals exposed to childhood abuse or traumas (3). In addition, social resilience, but not familial resilience, has been found to moderate the effect on depression in adults with a history of familial childhood abuse (26).

Despite the potential role of resilience as mediator between interpersonal depressive risk factors and hopelessness, previous studies did not consider this topic in depth even beyond the field of clinical depression (23). Therefore, the aim of this study is to investigate the putative role of resilience as a mediator between humiliation, adverse early family experiences, and hopelessness in a sample of depressed subjects. We previously reported that depression, resilience, humiliation, and adverse early family experiences are connected to each other. In particular, resilience has negative relation with humiliation, adverse early family experiences, and depression, showing a buffering effect in clinical and non-clinical samples (7, 27, 28). Therefore, we hypothesized that the relationships between adverse early family experiences and humiliation (as predictors) and hopelessness (as outcome) could be significantly mediated by resilience.

## Materials and Methods

### Participants

One hundred fifty consecutively admitted patients [70 males, 80 females; mean age 41.3 + 10.6 (SD); age range 19–64] for an index depressive episode were recruited from a psychiatry unit. All had a primary diagnosis of major depressive disorder (ICD-10, code F32) made by three senior psychiatrists (AR, FP, and MB). The patients were recruited at the admission and were currently being treated for depression with a homogeneous pharmacological treatment. No patients were treated with a psychotherapy approach. All patients have been hospitalized for a period of 10–14 days. The recruited patients were considered to be “severe cases” because they showed a score >2 in the depression subscale of the Brief Symptom Inventory, according to the results reported in a previous study based on an inpatient population (29). Patients with bipolar disorder, dementia, psychotic symptoms, and other diagnosis were excluded from the study. The questionnaires were administered to patients using a randomized presentation to avoid the order effect after 7 days from the admission. Eligible and available subjects provided written informed consent after receiving a complete description of the study and having an opportunity to ask questions. They then completed the self-report questionnaires in a light and quiet room. The average time to complete the questionnaires has been 23 minutes. The Ethics Committee for Medical Research of the University of L’Aquila (Italy) approved all recruitment and assessment procedures.

### Measures and Procedures

Hopelessness was assessed using the Italian version of the Beck Hopelessness Questionnaire (30). It consists of 20 items assessing the prevalence of hopeless thoughts in the past week (31). Participants rated their degree of hopelessness responding “True” or “False” to the questions (item example: “I can’t imagine what my life would be like in ten years”). Evidence of convergent validity of the scale has emerged from findings of negative associations with measures of hope (Hope Scale) and positive future thinking [Life Orientation Test; (32)]. The alpha reliability was 0.92.

Humiliation was assessed using the Italian version of the Humiliation Inventory (7). It consists of 32 items grouped in two dimensions: 20 items assessing the Fear of Humiliation and 12 items assessing the Cumulative Humiliation [item example: “At this point in your life, how much do your fear being scorned?”; (33)]. Participants rated their humiliation experiences on a 5-point Likert scale from 1 (not at all) to 5 (very much). We used the Humiliation total score only has been used and the alpha reliability was 0.97.

Early adverse family experiences were assessed using the Italian version of the Risky Family Questionnaire [RFQ; (34)]. It is composed of 13 items measuring the family background characterized by familial strife, a restrictive parenting style, and chaotic or neglectful parenthood [item example: “How often would you say there was quarreling, arguing, or shouting between a parent and you?”; (35)]. Participants rated aspects of their family environment on 5-point Likert scales ranging from 1 (not at all) to 5 (very often). Alpha reliability was 0.70.

Resilience was assessed using the Italian version of the Resilience Scale for Adult [RSA; (36)]. RSA is a 33 items scale measuring six factors of resilience and a total score (37), four personal factors (Perception of Self, Planned Future, Structured Style, and Social Competence) and two interpersonal factors (Family Cohesion and Social Resources). RSA total score only has been used for this study. Participants rated their resilience on 7-point semantic differential scales (item example: “My close friends and my family: appreciate my qualities □ □ □ □ □ □ □ despise my qualities”). The alpha reliability is 0.83.

### Data Analysis

Descriptive statistics were calculated for all variables considered. We also implemented a correlational analysis considering age and hopelessness and adverse early family experiences in order to explorer the hypothetical effect that adverse early family experiences can have in different phases of individual’s development. The relationship between humiliation and adverse early family experiences as independent variables, hopelessness as a dependent variable, and resilience as a mediating variable was assessed using a mediation approach with bootstrapping method in two different mediational models, one for each independent variable.

## Results

Descriptive statistics are reported in **Table 1**.

**Table 1:** Means and standard deviations of humiliation, hopelessness, risky family, and resilience (*N* = 150).

	Mean	SD
HI	2.56	1.10
BHS	0.51	1.17
RFQ	2.67	0.66
RSA	3.04	2.95

HI, Humiliation Inventory; BHS, Beck Hopelessness Scale; RFQ, Risky Family Questionnaire; RSA, Resilience Scale for Adult.

The above-mentioned correlational analysis (not tabulated analysis) showed that age did not significantly correlate hopelessness but slightly to RFQ (*r* = −0.21; *p* < 0.01).

**Figure 1:**
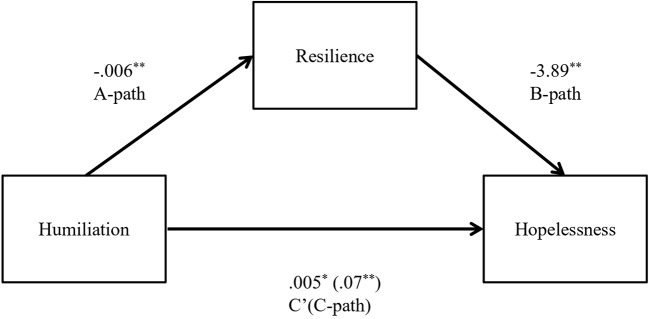
Indirect effect of Humiliation on Hopelessness through Resilience. Note. The unstandardized regression factors are reported. ^*^ *p* < 0.01, ^**^ *p* < 0.001.

In the first mediation model, humiliation was positively associated with hopelessness (B = 0.07, *t* (144) = 5.46, *p* = 0.001) and negatively related to resilience (B = −0.006, *t* (144) = −4.66, *p* = 0.001). Resilience was negatively associated with hopelessness (B = −3.89, *t* (144) = −5.14, *p* = 0.001). Because both the a-path and b-path were significant, mediation analyses were tested using the bootstrapping method with bias-corrected confidence estimates (38, 39). In the present study, the 95% CI of the indirect effects was obtained with 5000 bootstrap resamples (40). The mediation analysis confirmed that resilience mediated the relation between humiliation and hopelessness (B = 0.02; CI = 0.01 to 0.04). In addition, results indicated that the direct effect of humiliation on hopelessness slightly changed (B = 0.05, *t* (144) = 3.66, *p* = 0.003) when controlling for resilience, thus suggesting a small mediation effect. **Figure 1** displays the results of the first model.

**Figure 2:**
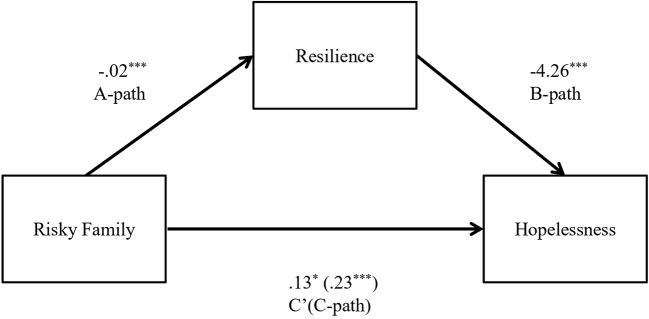
Indirect effect of Risky Family on Hopelessness through Resilience. Note. The unstandardized regression factors are reported. ^*^ *p* < 0.05, ^**^ *p* < 0.01, ^***^ *p* < 0.001.

In the second model, adverse early family experiences were positively associated with hopelessness (B = 0.23, *t* (144) = 4.20, *p* = 0.001) and negatively related to resilience (B = −0.02, *t* (144) = −4.44, *p* = 0.001). Resilience was negatively associated with hopelessness (B = −4.25, *t* (144) = −5.53, *p* = 0.001). As observed for the first model, both the a-path and b-path were significant. Results of the mediation analysis confirmed a mediating role of resilience in the relation between adverse early family experiences and hopelessness (B = 0.10; CI = 0.05 to 0.16). In addition, results indicated that the direct effect of adverse early family experiences on hopelessness changed (B = 0.13, *t* (144) = 2.41, *p* = 0.02) when controlling for resilience, thus suggesting a robust mediation. **Figure 2** displays the results of the second model.

## Discussion

To the best of our knowledge, this is the first study investigating the putative role of resilience as mediating variable between humiliation, adverse early family experiences (independent variables), and hopelessness (dependent variable) in a sample of depressed patients.

Hopelessness has been considered either a proxy measure of depression or an increased risk factor of suicidality or a moderator of some risk factors toward depression (41).

Adverse early family experiences represent a risk factor that is associated with different mental health disorders throughout the lifetime, including mood disorders, personality disorders, conduct disorders, and psychoses (13). Childhood trauma increases the risk of depression disorders, inappropriate emotion regulation (4), and violent behaviors (42).

Humiliation was also considered a risk factor for depression (7, 27), being associated with sense of entrapment, hopelessness, depression, and suicidal behavior.

Resilience is an ideal mediating variable that may influence the effects of risk factors to depressive symptoms (43, 44). Zimmerman et al. (45) hypothesized that resilience could operate through two main models: the compensatory model, in which resilience could additionally “neutralize” the effect of risk factors, acting independently from them, and the protective factor model in which resilience actually influences the effect of a risk factor interacting with it. In support of a “compensatory” effect of resilience toward depression, individuals with higher levels of resilience showed less psychopathological symptoms than individuals with lower levels of resilience (46). However, within a “protective factor model,” resilience could mediate the effect of negative life events towards psychosocial well-being (47).

Our results underlined the role of resilience as an “intervening variable” in determining the variance of hopelessness, but in different ways. In fact, resilience significantly and directly acts on hopelessness in both models, showing a robust mediation role only between adverse early family experiences and hopelessness. The same role has not been found between humiliation and hopelessness, where the mediation effect is smaller than the previous one. Within the model from humiliation to depressive symptoms, resilience seems not to influence the effect that humiliation exerts on the severity of depressive symptoms (e.g. hopelessness). In fact, resilience does not mitigate the effect of humiliation on hopelessness. On the other hand, resilience significantly acts as mediator between adverse early family experiences and hopelessness. These results confirmed a previous study, where a significant negative correlation between adverse early family experiences and resilience in a non-clinical sample was found, with resilience mediating between adverse early family experiences and the general severity symptoms (48). In the current study resilience displays a protective role between adverse early family experiences and hopelessness, not having the same “strength” between humiliation and hopelessness, thus maintaining a compensatory role. One of the reasons for that could be that resilience is mainly activated as a mediator when the independent variable (in this case adverse early family experiences) is strongly correlated to the dependent one (hopelessness). On the contrary, it does not happen between humiliation and hopelessness.

The effect of a confounding variable cannot be understood and studied only by a mediational model. Resilience itself has been addressed within a moderation framework as well. Although moderation and mediation approaches differ, they both underline the role of a “third intervening variable” in determining the variance of an outcome (i.e. dependent) variable (49). For instance, the moderating effect of resilience on suicidal ideation has been observed in patients with depression and/or anxiety disorders (50). Furthermore, resilience mitigates the development of depression during stressful life events (3, 51), coherently with a moderator function.

This study has important implications for a better comprehension of the importance of resilience, prevention, and treatment of interpersonal stressful factors in depression. In fact, protective factors such as resilience may become a potential target of psychological interventions (52, 53). At the same time, depressive symptoms, such as hopelessness, could negatively influence the patient’s outcome (54). Specifically, preventive interventions could improve the life and the resilient skills of children and avoid the development of depression and mental disorders in adulthood. The primary prevention includes efforts to prevent negative interpersonal events so that children grow up with less exposure to adversities. This prevention is focused on the change of the negative characteristics in the interpersonal environments. One example is the positive parenting program that is aimed to provide targeted education in order to support positive parenting (55). Secondary prevention includes interventions after a negative interpersonal event to reduce the immediate and short-term consequences. Interventions such as psychological first aid (PFA) implemented at school may provide the opportunity to identify at risk children early and to avoid severe psychopathology development (56). Finally, the tertiary prevention includes efforts to treat and reduce long-term consequences of negative interpersonal events. Psychotherapeutic interventions as EMDR are widely used for this goal (57). These two last preventive interventions include the improvement of personal and interpersonal resilient factors useful to counteract the negative consequences of interpersonal events (58).

The main limitations of the study are the cross-sectional approach and the use of self-report instruments. Moreover, the age range of the participants is large, and it has to be considered as a limitation. In fact, the adverse early family experiences could have different effects in different phases/ages of the individual’s development and that could influence the results of this study. However, several hypotheses could explain this relationship, but it is not related to the goal of the study. Finally, the lack of a personality assessment has to be considered as a limitation. It is conceivable that personality traits could influence resilience, the adverse past family and humiliation experiences. Furthermore, we use here resilience as unitary construct; therefore, a more refined analysis considering its components should be conducted in order to have an accurate assessment of the outcomes.

In conclusion, our study investigated the role of resilience as mediator variable between interpersonal factors (i.e. humiliation and adverse early family experiences) and depressive symptoms (i.e. hopelessness). Our results showed that resilience is a significant mediator overall between adverse early family experiences and hopelessness. Future research has to study the mediating role of resilience in depth in order to improve the prevention and the treatment of the negative consequences of interpersonal stressful factors in depression.

## Data Availability Statement

The datasets generated for this study are available on request to the corresponding author.

## Ethics Statement

The study was carried out in accordance with the recommendations of the Ministry of Health of Italy. All the procedures and the research project were approved by the Ethics Committee for Medical Research of the University of L’Aquila (Italy). All subjects gave written informed consent in accordance with the Declaration of Helsinki. The original ethics approval is available and can be submitted upon request.

## Author Contributions

AC made substantial contributions to study design, data acquisition, analyses, and interpretation of results; was involved in manuscript drafting and revision; gave final approval of the final version; and agreed to be accountable for all aspects of the work. PS made substantial contributions to study design and interpretation of results; was involved in manuscript drafting and revision. FP made substantial contributions to study design, analyses, and interpretation of results; was involved in manuscript drafting and revision. AR made substantial contributions to study design, analyses, and interpretation of results; was involved in manuscript drafting and revision. VSa made substantial contributions to study design, data acquisition, analyses, and interpretation of results; was involved in manuscript drafting and revision. MB made substantial contributions to study design, analyses, and interpretation of results; was involved in manuscript drafting and revision. DT made substantial contributions to data acquisition and was involved in manuscript drafting and revision. VSo made substantial contributions to data acquisition and was involved in manuscript drafting and revision. RR made substantial contributions to data acquisition, analyses, and interpretation of results; was involved in manuscript drafting and revision. All authors have read and approved the manuscript.

## Conflict of Interest

The authors declare that the research was conducted in the absence of any commercial or financial relationships that could be construed as a potential conflict of interest.
